# Study of Potential Blocking Peptides Targeting the SARS-CoV-2 RBD/hACE2 Interaction

**DOI:** 10.3390/ph17091240

**Published:** 2024-09-20

**Authors:** Sara M. Villada-Troncoso, Jenny Andrea Arévalo-Romero, Vanessa Hernández Rivera, Martha Pedraza-Escalona, Sonia M. Pérez-Tapia, Angela Johana Espejo-Mojica, Carlos Javier Alméciga-Díaz

**Affiliations:** 1 Institute for the Study in Inborn Errors of Metabolism—IEIM, Faculty of Science, Pontificia Universidad Javeriana, Bogotá 110231, Colombia; saravilladat@javeriana.edu.co (S.M.V.-T.); jennyarevalo@javeriana.edu.co (J.A.A.-R.); aespejo@javeriana.edu.co (A.J.E.-M.); 2 Instituto Distrital de Ciencia, Biotecnología e Innovación en Salud—IDCBIS, Bogotá 111611, Colombia; 3 Unidad de Desarrollo e Investigación en Bioterapéuticos (UDIBI), Escuela Nacional de Ciencias Biológicas, Instituto Politécnico Nacional, Prolongación de Carpio y Plan de Ayala S/N, Colonia Santo Tomás, Alcaldía Miguel Hidalgo, Mexico City 11340, Mexico; dhernandezr1708@alumno.ipn.mx (V.H.R.); sperezt@ipn.mx (S.M.P.-T.); 4 CONAHCyT-Unidad de Desarrollo e Investigación en Bioterapéuticos (UDIBI), Escuela Nacional de Ciencias Biológicas, Instituto Politécnico Nacional, Prolongación de Carpio y Plan de Ayala S/N, Colonia Santo Tomás, Alcaldía Miguel Hidalgo, Mexico City 11340, Mexico; martha.pedraza@udibi.com.mx

**Keywords:** SARS-CoV-2, COVID-19, blocking peptides, *Komagataella phaffii*, RBD variants

## Abstract

Background/Objectives: Severe acute respiratory syndrome coronavirus 2 (SARS-CoV-2), the causative agent of COVID-19, was declared a public health emergency in early 2020. The infection initiates when the receptor-binding domain (RBD) of the viral spike protein binds to human angiotensin-converting enzyme 2 (ACE2). Despite the success of vaccination efforts, the emergence of new variants highlights the ongoing need for treatments targeting these evolving strains. In silico methods previously identified peptides BP2, BP9, and BP11 as being capable of disrupting the RBD-ACE2 interaction, though their efficacy has not been experimentally validated until now. Methods: In this study, these peptides were recombinantly produced in the yeast *Komagataella phaffii*, and the activity was assessed in vitro using binding assays with multiple RBD variants and the inhibition of the RBD-ACE2 interaction. Results: The production yield for BP2, BP9, and BP11 was 14.34, 4.01, and 1.35 mg per culture liter, respectively. Noteworthy, the three BPs interacted with the RBD of SARS-CoV-2 variants of concern, with BP2 showing higher recognition. Finally, the BPs showed an RBD/hACE2 interaction blocking capacity with IC_50_ values between 1.03 and 5.35 nM, with BP2 showing the lowest values among the evaluated peptides. Conclusions: These results demonstrate that BP2, specifically, is a promising candidate for the development of novel therapeutic interventions targeting SARS-CoV-2 and other coronaviruses that use hACE2 for cellular entry.

## 1. Introduction

The coronavirus disease 2019 (COVID-19) is a highly contagious infectious disease of high importance in global public health. By August 2024, the World Health Organization (WHO) coronavirus disease dashboard reported that the disease resulted in more than 775 million infections and 7 million deaths (https://covid19.who.int/, accessed on 5 August 2024). It has mobilized global efforts to identify targets for treatments and vaccines. Human infection with severe acute respiratory syndrome coronavirus type 2 (SARS-CoV-2) can cause serious symptoms, including pneumonia, shock, severe acute respiratory syndrome, and multiple organ failure. Long-term reductions in lung function, arrhythmia, and death have been reported [1]. Although vaccines against COVID-19 provide strong protection against severe illness or death, cases continue to be reported, and new variants are constantly emerging (https://covid19.who.int/ accessed on 5 August 2024) [2,3]). In Colombia, over 6,364,636 cases have been reported, leading to 142,713 deaths, according to the Colombian National Institute of Health [4].

SARS-CoV-2 is an enveloped virus with a diameter of 120 nm belonging to the Betacoronavirus genus of the Coronaviridae family. The initial findings revealed that the virus interacts with the human angiotensin-converting enzyme 2 (hACE2) receptor [5]. hACE2 is a surface protein expressed in type II alveolar tissues and bronchial and nasal epithelial tissue, particularly in ciliated cells. However, it is also expressed in various human tissues. This protein recognizes the receptor-binding domain (RBD) present in the S1 subunit of the spike protein of the SARS-CoV-2 viral envelope [5,6]. This interaction represents the first step in viral entry, where the viral envelope fuses with the host–cell membrane. Consequently, this initial interaction has been identified as a primary target for the treatment of infected patients [1].

While COVID-19 vaccines have been highly effective in reducing disease severity and saving lives [7], the emergence of new variants capable of evading immune responses and treatments has been reported, even with a significant portion of the global population vaccinated [3,8]. After the first report of public health concern in 2019 in Wuhan, China, mutations have been detected throughout the SARS-CoV-2 genome. However, the more relevant seem to be those that appeared in the spike protein, as it affects both tropism and immune evasion [9]. In this regard, innovative molecular and cell-based therapeutics may help treat infected patients by eliminating the virus, alleviating the symptoms of acute respiratory distress syndrome and promoting tissue regeneration [1]. Next-generation treatments for COVID-19 include therapies based on the use of hACE2 decoys, T cells (including CAR-T), natural killer cells, extracellular vesicles, and mesenchymal/stromal cells [1]. Notably, these therapeutics may also contribute to preparedness for the next pandemic and to the development of novel treatments for other infectious diseases.

In addition, the impact of post-COVID-19 syndrome on patient health and well-being, as well as on healthcare systems and national economies, has underscored the urgent need for novel treatment strategies [10]. Persistent viral replication in reservoir tissues has been proposed as a key factor contributing to the development of long COVID, making the use of antiviral therapies a subject of ongoing research [10,11]. A recent publication from the PANORAMIC trial demonstrated that patients treated with molnupiravir reported enhanced well-being, fewer and less severe COVID-19 symptoms, reduced healthcare utilization, decreased absenteeism from work or study, and an overall improvement in quality of life compared to patients treated with the usual standard of care [12]. However, concerns about the mutagenicity of molnupiravir have emerged, highlighting the importance of continued investigation into novel therapeutic strategies for managing this condition.

Given the protein–protein interaction (PPI) that occurs for the initiation of the SARS-CoV-2 virus life cycle, the use of molecules that disrupt this interaction by occupying the available interaction sites has been proposed [13]. Candidates include neutralizing antibodies, small molecule inhibitors, and blocking peptides [14]. Unlike other molecules, blocking peptides have the advantage of higher selectivity and can be modified to target specific regions. Additionally, it has been proven that they are more effective in disrupting PPIs [15,16,17]. The in silico design of peptides based on the interaction between the RBD and hACE2 has proven to be an effective method of blocking virus entry [14,15,16]. Nevertheless, the design can be optimized to target the interface regions between the RBD of SARS-CoV-2 and hACE2 [18,19]. The resulting peptides may be highly specific while exhibiting broad antiviral activity.

Since the onset of the SARS-CoV-2 pandemic, bioinformatics has played a pivotal role in the identification and design of potential therapeutics against the virus [20,21,22]. These approaches have primarily involved the utilization of datasets for drug repurposing studies, employing techniques such as homology modeling, molecular docking, molecular dynamics simulations, quantitative structure–activity relationship (QSAR), and pharmacophore modeling. Additionally, the design of peptides has been a significant area of research [22]. Some examples of this area are the design of blocking peptides (BPs) from the hACE2 ligand-binding motif, targeting the RBD, as well as peptides derived from the heptad repeat (HR)1 and HR2 domains [22]. In Colombia, several studies have employed bioinformatics methodologies for the purposes of drug repurposing and peptide design [23,24,25,26,27]. In this regard, Ramirez and Gómez used a bioinformatics approach to design a set of blocking peptides, BP2, BP9, and BP11, which were derived from the hACE2 regions that interact with the SARS-CoV-2 RBD, which may bind to the RBD and inhibit the virus’s entry into cells [28]. Briefly, the design of the BPs was based on the molecular study and understanding of the RBD/hACE2 interaction, using structures deposited in the Protein Data Bank. Relevant regions for the interaction, along with surrounding amino acids, were identified and selected. Connecting amino acid sequences were used to link the ACE2 interface regions that interact with the RBD of the SARS-CoV-2 spike protein. The 3D structures of BP candidates were modeled and structurally aligned with models published in the PDB (e.g., 6VXX, 6LZG, 6VSB, and 6M0J) for an assessment of their potential interaction with the RBD. Proteins that passed this screening underwent molecular dynamics and were finally analyzed to estimate their interactions. Based on the interaction free energy, dissociation constant, and in silico solubility, the best characteristics were selected as the BP candidates [28]. The authors proposed that these BPs may have a higher affinity for the RBD than hACE2 since they have lower theoretical *K*_d_ values (between 1.3 × 10^−9^ and 1.9 × 10^−10^ M) than those experimentally determined for the RBD-hACE2 interaction (3.8 × 10^−9^ M) [28]. However, the experimental evaluation of these BPs has not yet been reported, and the modeling did not account for the presence of post-translational modifications, such as N-glycosylations, which could affect the binding [29].

We have previously used the yeast *Komagataella phaffii* (formerly known as *Pichia pastoris*) as a successful platform to produce several recombinant proteins, including therapeutic enzymes [30,31,32,33,34,35], enzymes with industrial applications [36,37], and a bovine vaccine candidate [38,39]. In this study, we extended our experience with the yeast *K. phaffii* to express the BP2, BP9, and BP11 peptides and evaluate their capacity to block the interaction between SARS-CoV-2 RBD and hACE2 in vitro. These results may contribute to the ongoing efforts to develop effective therapeutic interventions against SARS-CoV-2. By targeting the critical interaction between the viral RBD and hACE2, the recombinant peptides evaluated in this research offer a promising treatment alternative for COVID-19. The in vitro validation of the blocking peptides, particularly BP2, which demonstrated superior binding affinity and inhibitory potency, underscores the potential of these peptides in combating current and future variants of concern. These findings not only reinforce the applicability of bioinformatic design methods in drug discovery but also provide a foundation for developing next-generation antiviral strategies that can mitigate the evolving challenges posed by SARS-CoV-2 and other infectious agents.

## 2. Results

### 2.1. Peptide Properties and hACE2 Interaction Modeling

**Figure 1:**
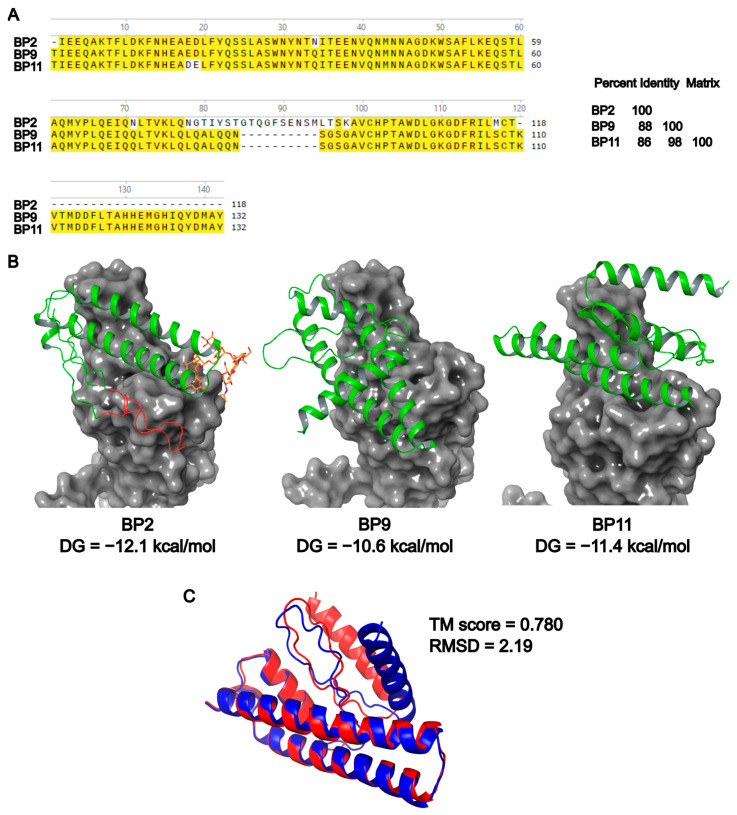
Bioinformatic analysis of blocking peptides. (**A**) Protein sequences alignment and percent identity matrix for BP2, BP9, and BP11. Multiple sequence alignment was performed by using Clustal Omega [40]. Amino acids within the box at the N-terminal represent those present in other blocking peptides [14] (see Section 3) (**B**) Protein structure modeling of BP2, BP9, and BP11 (green) and prediction of the interaction with SARS-CoV-2 RBD (gray). Additional coil present in BP2 is colored in red. Modeling of BPs and RBD (derived from PDB 6m0j) interaction was carried out by using the AlphaFold server. A high mannose N-glycan chain (orange) was included in BP2 to simulate a high mannose glycan synthesized by *K. phaffii*. (**C**) Protein structure alignment of BP9 (blue) and BP11 (red) was carried out by using the TM-align server.

BP sequences were obtained from the patent application of Ramírez and Gómez [28]. The sequences of the blocking peptides showed a percentage identity ranging from 86.1% to 98.5%, with BP2 having the lowest identity compared to BP9 and BP11 (Figure 1A). This difference was primarily due to the presence of an additional coil in BP2 (shown in red in Figure 1B) and a longer C-terminal sequence in BP9 and BP11 compared to BP2. Based on these sequences, we predicted the physicochemical properties of BP2, BP9, and BP11 (Table 1 and Supplementary Table S1). BP2 is the shortest peptide, whereas BP9 and BP11 share identical sequences except for a single amino acid inversion (i.e., ED/DE). GRAVY values predicted the presence of hydrophilic structures for all peptides, which were mostly α-helices, similar to H1 of the human protein hACE2.

Figure 1B shows the modeled structure for the three peptides, as well as the interaction with SARS-CoV-2 RBD. Although comparable outcomes can be achieved using experimentally resolved structures, the results may be limited by several factors, including the absence of linkers in the structures available in the PDB structures but present in the BPs. Ramírez and Gómez [28] employed the Phyre2 server to model peptides and performed structural alignments against PDB entries to assess interactions during the design of the blocking peptides. However, this method did not account for N-glycosylation on BP2, which resulted in lower binding predictions for BP2 that were not supported by the experimental data from our study. To address this, the latest version of AlphaFold was utilized to model not only the peptide structures but also their interactions with the RBD, providing a more comprehensive and accurate prediction.

All peptides were predicted to have α-helix and coils. Although the peptides share a high sequence identity, they were predicted to interact differently with RBD. In this regard, BP2 showed the highest predicted affinity, followed by BP11 and BP9. Additional data from bioinformatic analysis on BPs-RBD interactions are included in Supplementary Table S2. Additionally, it was observed that the N-glycan present in BP2 might play a crucial role in its interaction with RBD. Protein structure alignment of BP9 and BP11 showed that the ED/DE inversion in BP11 might induce a change in the protein structure, as indicated by a change in the TM score (0.783 vs. 1.0 for the BP9/BP9 or BP11/BP11 alignments) and a predicted RMSD of 2.19 Å (Figure 1C).

### 2.2. Transformation of K. phaffii NRRL Y-11430

**Figure 2:**
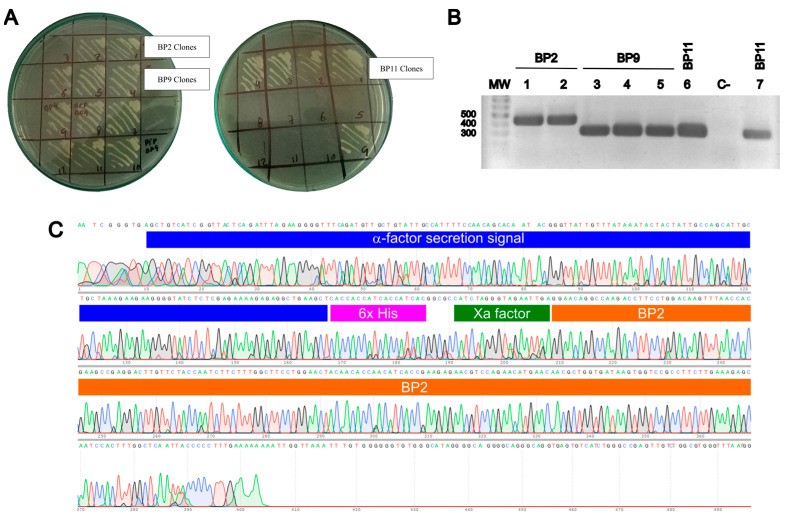
*K. phaffii* transformation and molecular confirmation. (**A**) *K. phaffii* NRRL Y-11430 was transformed with pPICZα-A plasmids expressing BP2, BP9 and BP11. Transformed clones were selected by zeocin resistance. (**B**) Molecular confirmation of *K. phaffii* BP2, BP9 and BP11 clones by the amplification of a region of the plasmid to confirm the presence of the target gene. PCR product size: BP2 = 427 bp, BP9 and BP11 = 307 bp. C-: PCR negative control. (**C**) Expression cassette was confirmed via DNA sequence. Representative result of a BP2 clone showing the presence of the α-factor secretion signal, 6x-His tag, Xa factor, and a fragment of the BP2 sequence. Similar results were obtained for BP9 and BP11 clones (Supplementary Figure S1).

The successful transformation of yeast cells with the expression plasmids for BP2, BP9, and BP11 was determined by the growth of colonies in the presence of zeocin at both 100 and 200 µg/mL (Figure 2A). After transformation, clones of BP2, BP9, and BP11 clones were selected for molecular confirmation through the amplification of a plasmid region to confirm the presence of the target gene. As observed in Figure 2B, the agarose gel electrophoresis of the colony PCR demonstrated the presence of the expected amplicon in most of the evaluated colonies. Amplicons were sequenced to confirm the presence of the expression cassette. Figure 2C shows the electropherogram and the regions of the expression cassette to which they correspond for a BP2 clone. Similar results were obtained for the BP9 and BP11 clones (Supplementary Figure S1).

### 2.3. Screening of High-Expression Clones

**Figure 3:**
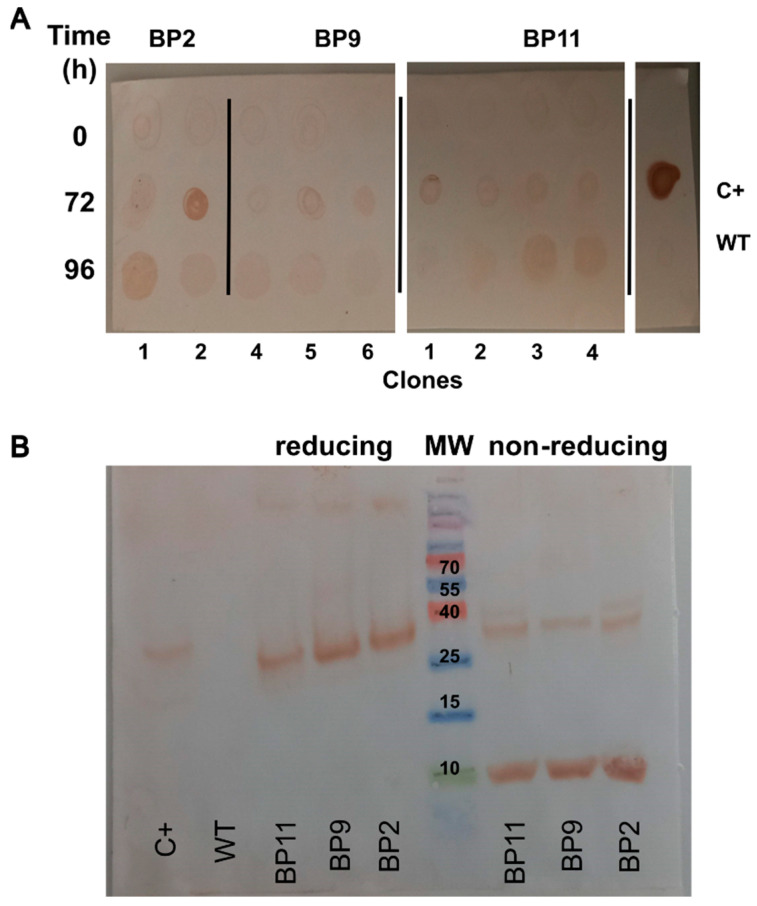
Screening of *K. phaffii* clones transformed with pPICZα-A plasmids expressing BP2, BP9 and BP11. (**A**) Expression of the blocking peptides was evaluated in randomly selected clones at 100 mL. BPs were followed up by dot blot using an anti-His tag antibody. (**B**) Western blot of BPs from the crude extract of *K. phaffii* BP2, BP9, and BP11 clones. Samples were evaluated under reducing and non-reducing conditions by using an anti-His tag antibody. C+: Positive control of a recombinant lipase produced in *E. coli* (a kindly gift of Dr. Gina López, USBA, Pontificia Universidad Javeriana).

Molecularly confirmed clones of *K. phaffii* NRRL Y-11430 for BP2, BP9, and BP11 were selected for the evaluation of their ability to produce the BPs. The screening was conducted by inducing the production of recombinant peptides at a 100 mL scale. Samples were collected at 24 h intervals to monitor peptide production by dot blot against the 6x-His tag. Samples collected after 72 and 96 h of induction showed a marron spot, indicating the production of the recombinant BPs in all of the evaluated clones (Figure 3A). Meanwhile, the non-transformed strain of *K. phaffii* NRRL Y-11430 did not produce any signal. The qualitative dot blot assays identified BP production in clones 1 and 2 of BP2, clone 2 of BP9, and clones 4 and 5 of BP11.

The initial characterization of the recombinant BPs was performed via protein electrophoresis (SDS-PAGE) and protein identification via immunodetection (Western blot) of the 6x-His tag. In the Western blot (Figure 3B), multiple intense bands were observed on the sample lanes. The presence of a band with a molecular weight between 25 and 40 kDa under non-reducing conditions was observed. Additionally, under reducing conditions, two predominant bands were identified. The first band appeared between 25 and 40 kDa, and the second band was approximately 10 kDa. However, bands of higher molecular weight were also observed. No bands were detected in the negative control (non-transformed *K. phaffii*). These results suggest that the BPs may be produced both as monomers and as protein aggregates (i.e., dimers and trimers).

### 2.4. Scaling Up, Production, and Purification of Recombinant BPs

**Figure 4:**
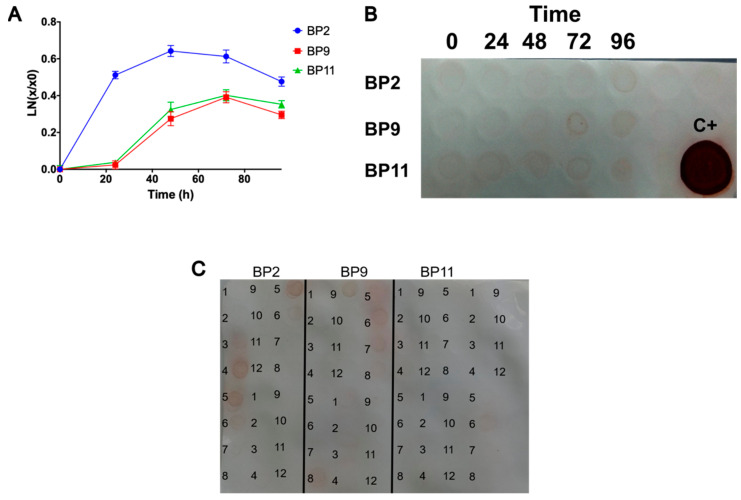
Production and purification of BPs at 400 mL. (**A**) Quantification of biomass during 96 h of induced heterologous peptide production of BP2, BP9, and BP11. Results are presented as mean ± SD. (**B**) Immunodetection of the BPs, in the crude extract, through their 6x-His tag. C+: Positive control of a recombinant lipase produced in *E. coli*. (**C**) Dot blot assay of the eluted fractions from the purifications.

Considering the above results, clones 1, 5, and 4 for BP2, BP9, and BP11, respectively, were selected for scaling up to 400 mL. The biomass was estimated during the induction process, as illustrated in Figure 4A. Both BP9 and BP11 demonstrated an increase in biomass production between 24 and 48 h, which remained stable during the remainder of the induction period. In contrast, BP2 exhibited a pronounced increase in biomass during the initial 24 h of induction, followed by a modest further increase at 48 h. Thereafter, a gradual decline in biomass was observed. As illustrated in Figure 4B, the evaluated clones produced recombinant BPs with the highest levels at 72 and 96 h of induction.

At 400 mL, the crude extracts were subjected to nickel affinity chromatography-based purification of the BPs. The findings indicated that recombinant BPs were predominantly present in the initial purification fractions, with BP2 and BP9 exhibiting the most prominent signals (Figure 4C). After affinity purification, the BPs were subjected to desalting to remove the imidazole before protein quantification. The results showed that 0.118, 0.080, and 0.132 mg/mL of total protein were obtained for BP2, BP9, and BP11, respectively. However, these results contrast with those in the dot blot assay, where BP11 showed the lowest production of the recombinant peptide, suggesting that some interferences were still present within the sample.

**Figure 5:**
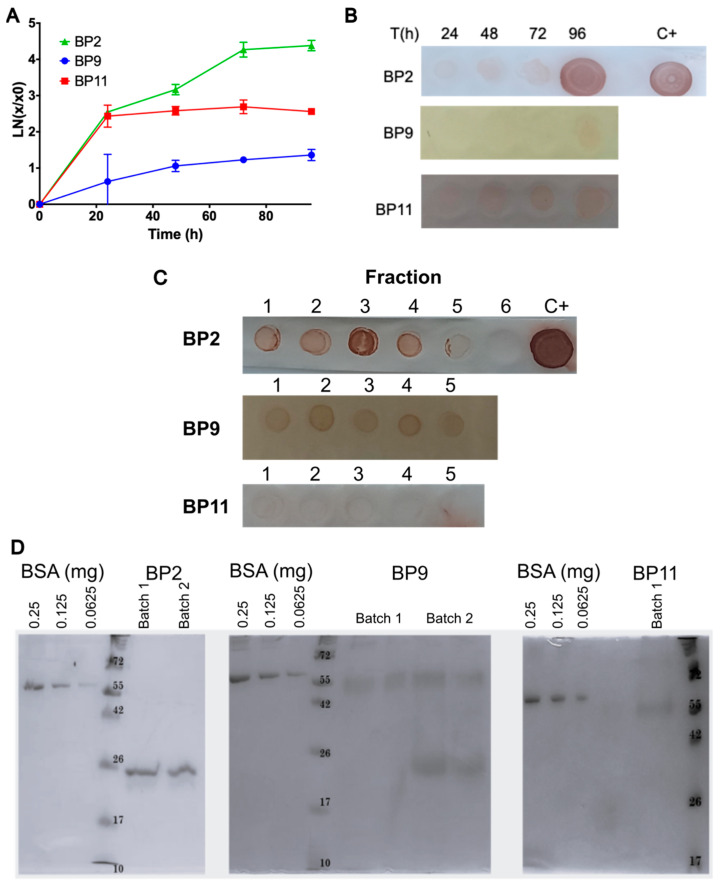
Production and purification of BPs at 1.65 L. (**A**) Quantification of biomass during 96 h of induced heterologous peptide production of BP2, BP9, and BP11. Results are presented as mean ± SD. (**B**) Immunodetection of the BPs, in the crude extract, through their 6x-His tag. C+: Positive control of a recombinant lipase produced in *E. coli*. (**C**) Dot blot assay of the eluted fractions from the purifications. (**D**) SDS-PAGE of purified BP2, BP9 and BP11. Two different batches were evaluated for BP2 and BP9. A BSA curve between 0.0625 and 0.25 mg was included for densitometry quantification.

The production of recombinant BPs was then scaled to 1.65 L in a fed-batch culture, following previously standardized protocols [31,33,35]. During the production process of BPs, biomass was monitored (Figure 5A) and BP production was confirmed through dot blot (Figure 5B) in samples taken every 24 h during the 96 h of induction. As observed at the 400 mL scale, the BP2 clone showed the highest cell growth compared to BP9 and BP11. Immunodetection indicated that the BPs were produced at this scale, with the highest results observed by the end of the induction phase (>72 h) and BP2 showed the highest production. The same process used for the purification of the 400 mL culture was employed for the 1.65 L culture, beginning with clarification processes via filtration and then affinity chromatography (see Supplementary Figure S2), followed by desalting. As illustrated in Figure 5C, the dot blot assay of the purification fractions for BP2, followed by BP9 and BP11, showed increased signal intensities, confirming the enhanced production of BP2 at this scale. Table 2 presents the final quantification of BPs and the production yield after purification. BP2 exhibited higher total protein production (23.66 mg) compared to BP9 (6.62 mg) and BP11 (2.23 mg). Similarly, BP2 demonstrated higher productivity values compared to BP9 and BP11. Similar to the results at the 100 mL scale, the analysis of the purified proteins via SDS-PAGE (Figure 5D) showed that BPs were present as dimers (BP2 and BP9) and trimers (BP9 and BP11).

### 2.5. Binding and Blocking Capacity of Recombinant BPs against the RBD of Diferent SARS-CoV-2 Variants

**Figure 6:**
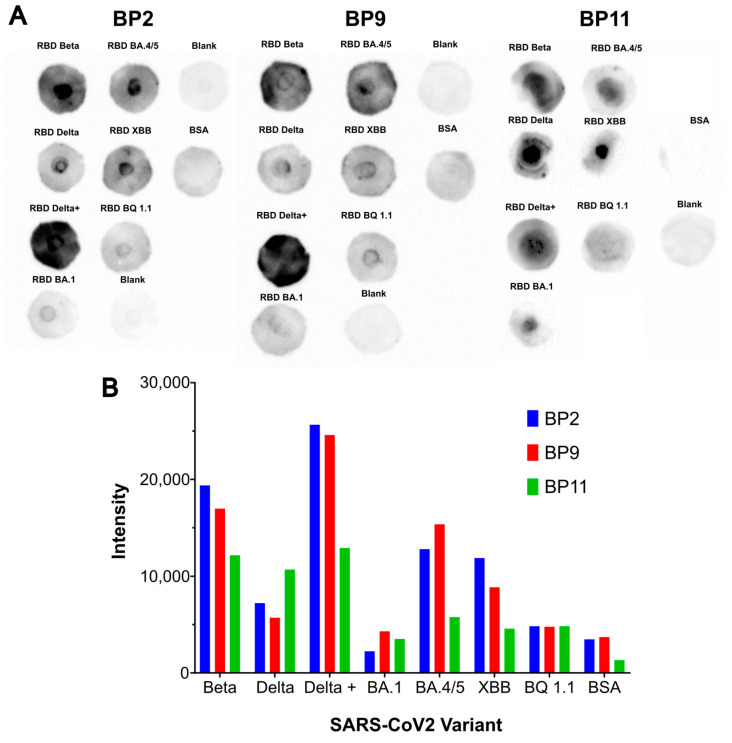
Evaluation of BPs binding to RBD from different SARS-CoV-2 variants of concern. (**A**) Dot blot assay for RBD/hACE2 interaction. BP samples were allowed to interact with Beta, Delta, Delta+, BA.1, BA.4/5, XBB, and BQ1.1 RBDs. (**B**) Densitometric quantification of BPs binding to SARS-CoV-2 RBD variants of concern. Densitometry was carried out by using ImageJ. BSA: negative control; Blank: BP without RBD adding.

The purified proteins produced at 1.65 L were used to evaluate binding to the RBD of the SARS-CoV-2 variants of concern Beta, Delta, Delta+, Omicron BA.1, Omicron BA.4/5, Omicron XBB, and OmicronBQ1.1 (Figure 6A). The dot blot showed that the three BPs interacted differently with the evaluated SARS-CoV-2 RBD variants.

To analyze the binding of BPs to the principal SARS-CoV-2 RBD variants of concern, the signal intensity was measured using ImageJ 1.53a. As shown in Figure 6B, BP2 exhibited higher recognition to Beta, Delta+, and XBB variants compared to the other BPs. BP9 showed higher recognition affinity than BP2 and BP11 only for the BA.4/5 variant, while BP11 showed higher affinity recognition for the Delta variant, compared to BP2 and BP9. Overall, the highest affinities were observed for the variants more similar to the Wuhan RBD (i.e., Betta, Delta, and Delta+), which had fewer mutations in the RBD. In contrast, Omicron-derived variants (i.e., BA.1, BA4/5, XBB, and BQ 1.1), which exhibit high mutagenicity on the RBD, demonstrated low affinities. Notably, no binding was observed under the condition of the experiment for variant BA.1 for any of the evaluated BPs.

**Figure 7:**
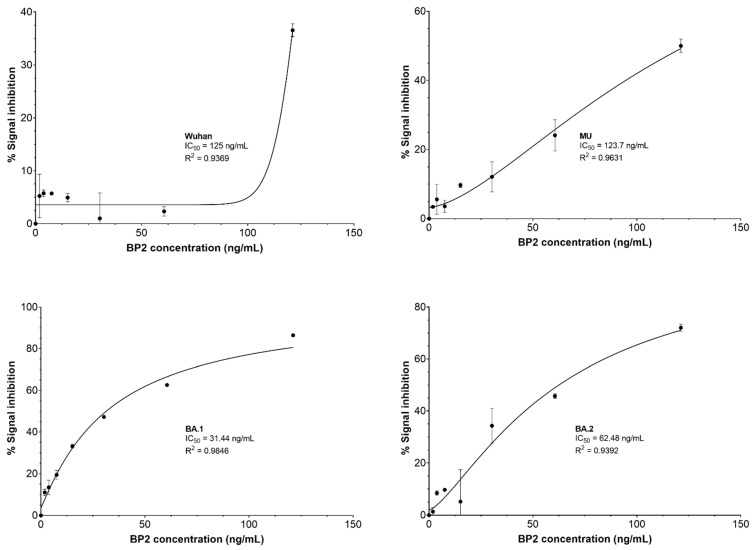
Blocking assay for BP2. The percentage of signal inhibition was estimated using the cPass kit according to the manufacturer’s instructions. BP2 concentrations of 121.17 ng/µL, 60.59 ng/µL, 30.29 ng/µL, 15.15 ng/µL, 7.57 ng/µL, 3.79 ng/µL, and 1.89 ng/µL were evaluated against the RBD SARS-CoV-2 variants Wuhan, MU, Omicron BA.1, and BA.2. IC_50_ was estimated by using nonlinear regression.

**Figure 8:**
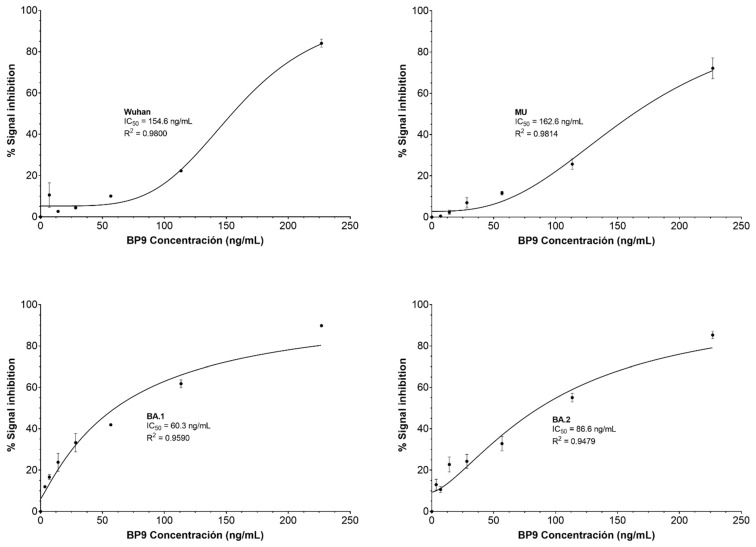
Blocking assay for BP9. The percentage of signal inhibition was estimated using the cPass kit according to the manufacturer’s instructions. BP9 concentrations of 226.80 ng/µL, 113.40 ng/µL, 56.70 ng/µL, 28.35 ng/µL, 14.20 ng/µL, 7.08 ng/µL, and 3.54 ng/µL were evaluated against the RBD SARS-CoV-2 variants Wuhan, MU, Omicron BA.1., and BA.2. IC_50_ was estimated by using nonlinear regression.

The blocking of the RBD/hACE2 interaction by the BPs was assessed through a neutralization ELISA assay and the calculation of the inhibitory concentration (IC_50_). Due to the binding results and the low protein yield, BP11 was not selected for this assay. BP2 exhibited IC_50_ values of 125, 123, 32.44, and 62.48 ng/mL for the RBD of Wuhan, MU, Omicron BA.1, and BA.2 variants, respectively (Figure 7). In contrast, BP9 exhibited IC_50_ values of 154.6, 162.6, 60.29, and 86.60 ng/mL for the RBD of Wuhan, MU, Omicron BA.1, and BA.2 variants, respectively (Figure 8). Overall, BP2 exhibited lower IC_50_ values than BP9 for all the evaluated variants.

## 3. Discussion

Even though the global COVID-19 pandemic was officially over by May of 2023, its etiological agent, SARS-CoV-2, remains of great interest in public health and in virology for its unprecedented rates of mutagenicity in such a brief period. Vaccines have played a significant role in protecting a substantial portion of the global population from SARS-CoV-2 infection and subsequent mortality. However, the emergence of multiple variants has led to the persistence of infection across the globe. Some of these variants have evolved to evade the effects of antiviral treatments [41,42,43]. Consequently, the development of novel therapeutic strategies is a critical area of research. Peptides are at the forefront of the modern search for novel, potent, selective, and safe therapeutics. Their remarkable target specificity and potency, often demonstrated by EC_50_ values in the nanomolar range, underscore their potential as cutting-edge treatments, as shown in this study. Peptides have significantly reshaped the pharmaceutical landscape by offering solutions to challenges that traditional small molecules often cannot overcome, such as the global rise in viral infections [44]. For instance, multiple sarbecoviruses have triggered major human outbreaks over the past two decades, highlighting the critical need for pandemic preparedness. This emphasizes the importance of developing therapies that specifically target the interaction between these viruses and their hACE2 receptor [45]. Notably, the design of peptides, like those explored in this study, paves the way for broad-spectrum therapeutic options capable of addressing a wide range of viral targets. Currently, peptides represent over 5% of the global pharmaceutical market, valued at USD 42.05 billion, further cementing their role as a pivotal element in the future of antiviral drug development [44].

One of the first steps in the infection process is the interaction of the SARS-CoV-2 RBD with the human receptor hACE2. This interaction is essential for the attachment and subsequent entry of the virus into human cells [46,47,48,49]. Molecules such as antibodies and small molecules have been evaluated as protein–protein interaction inhibitors [50,51,52]. However, peptides designed and optimized through in silico approaches have been demonstrated to be potent and broader inhibitors [15,16,44]. Most of the evaluated peptides are short sequences (i.e., 6–85 residues) [15,16,53], such as those designed by Mahmoudi Azar et al. [18] and Karoyan et al. [54]. Moreover, these peptides were chemically synthesized with a minimal number of residues. In this study, we evaluated recombinantly produced blocking peptides (BPs) with 134 to 148 amino acids to block the RBD/hACE2 interaction. The biological synthesis of peptides, as opposed to chemical synthesis employed in other studies, enables the production of longer amino acid chains that can target multiple regions of the protein–protein interactions, mimicking the broader binding capability of antibodies. Furthermore, biological synthesis allows for the incorporation of post-translational modifications, such as N-glycosylation, which was crucial for the blocking activity of BP2 and is less immunogenic compared to chemically introduced glycosylation [55].

Although all three BP genes were successfully cloned into *K. phaffii*, and the recombinant peptides were produced and purified, marked differences in production among these BPs were observed. At the bioreactor scale, BP2 production was 2.3- to 3.5-fold higher than that of BP9 and 10.6-fold higher than that of BP11. The *Saccharomyces cerevisiae* α-mating factor (MF) is the most common and widely used signal sequence for the secretion of recombinant proteins in *K. phaffii*. This signal consists of 85 amino acids divided into two regions: a 19-amino acid pre-peptide at the N-terminus, which acts as a signal peptide, and a 66-amino acid pro-peptide at the C-terminus, which facilitates protein secretion. The pre-peptide directs secretory proteins to the endoplasmic reticulum (ER), while the pro-peptide enhances secretion efficiency by mediating the transport of secretory proteins into ER-derived COPII vesicles [56,57]. Once inside the ER, recombinant proteins undergo post-translational modifications such as N-linked glycosylation, which has been shown to affect secretion efficiency in *K phaffii.* The proteins are then transported to the Golgi apparatus (GA), where they may undergo additional modifications. Finally, secretory vesicles transport mature proteins to the plasma membrane, where they are secreted into the extracellular milieu. This efficient secretion process is crucial for producing high-quality and high-yield recombinant proteins in *K. phaffii* [58,59].

BP2 has three potential N-glycosylation sites at asparagine (Asn) 33, 70, and 77. However, the analysis with NetNGlyc-1.0 (Supplementary Figure S3) [60] predicted a higher probability of N-glycosylation in Asn33. In contrast, no sites for N-glycosylation were predicted for peptides BP9 and BP11 (Supplementary Figure S3). Since N-glycosylation helps to increase structural stability and solubility and serves as a quality control mechanism by evading the unfolded protein response (UPR) pathway activated in the ER [61], the absence of this N-glycosylation can make BP9 and BP11 not only more prone to aggregation but also promote their degradation through ER and Golgi-mediated quality control mechanisms [62]. In this regard, the lack of N-glycosylation in BP9 and BP11 could impact their processing and stability, possibly leading to a reduction in their production.

Furthermore, differences in BP9 and BP11 production may be associated with the Asp-Glu inversion present in BP11 compared to BP9 (Figure 1). Although the exact impact of this inversion on protein structure remains unknown, an assessment of the similarity of BP9 and BP11 structures using the TM score server [63] predicted that the Asp-Glu inversion in BP11 caused a change in the protein structure, as indicated by the TM score (0.780 vs. 1.0 for the BP9/BP9 or BP11/BP11 alignments) and a predicted RMSD of 2.19 Å. Overall, while N-glycosylation benefits BP2 production, the lower productivity of BP9 and BP11 is likely related more to folding issues than to glycosylation, considering that the ER and GA pathways involve various post-translational modifications beyond N-glycosylation. In addition, although the clones with the highest production levels were selected for each of the BPs, and multicopy clones were chosen, it is possible that these differences are also due to differences in gene expression. Purification conditions using affinity chromatography for the 6x-His tag allowed for successful separation from other proteins present in the culture, but molecular weight analysis after purification suggested that the conditions used in this study may favor aggregate formation, as the final peptides were mostly present as dimers for BP2 and trimers for BP9 and BP11.

Regarding the affinity to RBD and the blocking of the RBD/hACE2 interaction, the dot blot assay showed that BP2 exhibited the highest affinity among all evaluated peptides with most of the RBD variants. Higher affinities were observed for variants with a fewer number of mutations in the RBD, such as Betta and Delta+, compared to the Omicron variants like BA.1, BA.4/5, XBB, and BQ1.1, which have multiple mutations in the RBD that may affect the ability of the BPs to interact with the target molecule [41,42,64,65,66]. In contrast, when the BPs were evaluated using the ELISA cPass assay, lower IC_50_ concentrations were observed for Omicron variants compared to Wuhan and MU variants. These results suggest that dimeric and trimeric conformations of the peptides may be important for the activity of the BPs across multiple RBD variants. Considering the viral S protein is a trimeric structure [46,67], the interaction with trimeric conformation of the BPs may increase the probability of interaction with the three stable regions targeted by the peptides. The binding results and IC_50_ values contrast with those obtained by Ramírez and Gómez [28], as they predicted that BP11 may have a higher affinity (∆G −14.2 kcal/mol, *K*d 9.4 × 10^−11^ M) than BP2 (∆G −12.6 kcal/mol, *K*d 1.3 × 10^−9^ M) and BP9 (∆G −13.7 kcal/mol, *K*d 1.9 × 10^−10^ M). Nevertheless, the binding results and IC_50_ values agree with bioinformatic analysis performed in the present study, which predicted that BP2 may have a higher affinity for hACE2 than BP9 and BP11 (Figure 1B). The discrepancy between these outcomes may be attributed to the software used for protein structure modeling and protein–protein interaction, as well as the incorporation of the N-glycan in BP2. In this regard, it has been demonstrated that N-glycosylation plays a pivotal role in protein–protein interaction [68,69].

Recent reports of VOC highlight Omicron variants as the most present in the global population. Omicron variants have reduced severity in terms of of clinical presentations but exhibit higher transmission rates [9,42,65,66]. Interaction and blocking assays with the BPs determined that even when qualitative affinity seemed low, the BP concentration needed for blocking the interaction between Omicron RBDs and hACE2 was lower than with previous variants. This demonstrates that the presence of additional mutations in these variants did not prevent the inhibition of interaction and establishes the BPs as an alternative treatment for multiple variants, with potential efficacy even against new variants that could emerge.

Previous reports have evaluated peptides for inhibiting the SARS-CoV-2 interaction with hACE2. Notably, most of these peptides share a consensus sequence that is also present in the BPs evaluated in this study (Figure 1A, black box), suggesting that it is an important sequence to mediate their binding to the SARS-CoV-2 RBD. Regarding the blocking capacity of previously reported peptides, Odolczyk et al. reported IC_50_ concentrations between 5 and 10 mM for a peptide of six residues [19]. Karoyan P et al. reported three peptides with IC_50_ values between 42 and 53 nM, which were evaluated against the SARS-CoV-2 (2019-CoV) variant [54]. In this context, the current literature for blocking peptides reports IC_50_ values mainly within the range of nM and mM concentrations [15,44,52]. Considering the IC_50_ results of the peptides evaluated in this study, and their predicted molecular weights, the IC_50_ values for BP2 and BP9 are Wuhan 4.11 nM, 3.39–5.09 nM, MU 4.07, 3.57–5.35 nM, BA.1. 1.03 nM, 1.32–1.98 nM and BA.2: 1.32 nM, 1.9–2.85 nM. Notably, the results showed that both BP2 and BP9 have IC_50_ values in the nM range, which are similar or even better to those reported for other SARS-CoV-2 blocking peptides, highlighting the potential of these BPs for the developing of a novel treatment strategy for COVID-19. On the other hand, antibodies that have been approved for COVID-19 treatment have been reported to effectively inhibit in the pM range [70,71]. However, it has also been reported that the SARS-CoV-2 variant may acquire resistance to these antibodies in concentrations of up to 50 µg/mL. The emergence of Omicron variants has prompted the FDA to limit the use of the bamlanivimab and etesevimab cocktail due to its loss of efficacy [50].

In summary, the binding and IC_50_ results of BP2 and BP9 peptides have significant implications for the development of treatments for COVID-19. BP2 demonstrated the highest affinity for various SARS-CoV-2 RBD variants and exhibited lower IC_50_ values compared to BP9, indicating its superior efficacy in inhibiting the RBD/hACE2 interaction, which is essential for viral entry into human cells. These characteristics suggest that BP2 has the potential to serve as an effective therapeutic agent, offering an efficient treatment option against multiple SARS-CoV-2 variants, including those with significant mutations that have demonstrated resistance to other therapies. The ability of BP2 to maintain high binding affinity and potent inhibitory capacity across diverse RBD variants highlights its value as a promising candidate for further development, contributing to the broader arsenal of antiviral strategies needed to address the ongoing and future challenges posed by SARS-CoV-2 and related coronaviruses.

## 4. Materials and Methods

### 4.1. Blocking Peptide Information and Protein–Protein Interaction Modeling

BP sequences were retrieved from Ramírez and Gómez (Figure 1A, [28]). The physicochemical properties of BP2, BP9, and BP11 were predicted using Expassy ProtParam [72], whereas N-glycan sites were predicted using NetNGlyc 1.0 server [60]. The modeling of BPs and SARS-CoV-2 RBD (derived from PDB 6m0j interaction was carried out by using the AlphaFold server [73]. A high mannose N-glycan chain was included in BP2 using the AlphaFold server to simulate a high mannose glycan synthesized by *K. phaffii* [74]. Protein structure alignment was carried out by using the TM-align server [63]. The binding energy of BPs-hACE2 interactions was predicted using the web server PRODIGY [75].

### 4.2. Expression Vectors

The DNA sequences of BPs were optimized for *K. phaffii* expression, synthesized, and cloned into pPICZα-A (Thermo Fisher Scientific Inc., Waltham, MA, USA) using Gene Universal (Newark, NJ, USA). The plasmids enabled the production of a recombinant BP with a 6x-His tag sequence placed at the N-terminal. The expression cassettes were inserted downstream of the *AOX*1 promoter and the α-factor secretion signal. The pPICZα-A plasmid contains a BleoR resistance gene that allows for the selection of *E. coli* and *K. phaffii* transformants by zeocin. pPICZα-BP plasmids were kindly provided by Dr. César Ramírez from Unidad de Ingeniería Celular y Molecular (UICyM) at the Instituto Distrital de Ciencia, Biotecnología e Innovación en Salud—IDCBIS, Bogotá, Colombia.

### 4.3. K. phaffii Transformation

The recombinant plasmids were linearized to transform *K. phaffii* NRRLY-11430 competent cells via electroporation. Plasmid Linearization was performed using restriction enzyme *Pme*I (New England Biolabs, Ipswich, MA, USA), which has a single cutting site in the p*AOX*1 region of the plasmid. The enzymatic reaction was incubated for 3 h at 37 °C, followed by inactivation at 65 °C for 20 min. The DNA was precipitated by applying 5 µL of potassium acetate 5 M, pH 4.8 and 100 µL of isopropanol. The linearized DNA was resuspended in 10 µL of distilled water. The competent cells were prepared in accordance with the protocol outlined in the Easy Select Pichia Expression Kit (Thermo Fisher Scientific Inc., Waltham, MA, USA). In brief, a 1 mL vial of the conserved *K. phaffii* NRRLY–11430 was cultured in 5 mL of YPD broth [1% (*w*/*v*) yeast extract, 2% (*w*/*v*) peptone, 2% (*w*/*v*) glucose] at 30 °C and 250 rpm for 16 h. Subsequently, the culture was used to inoculate 500 mL of YPD broth, which was then cultured under the previously described conditions until an optical density (OD_610nm_) of between 1.3 and 1.5 was reached. The culture was centrifuged for 5 min at 15,000 rpm and 4 °C and washed twice with sterile water. Finally, the cells were then resuspended in ice-cold 1 M sorbitol. The competent cells were then utilized immediately. The transformation of the competent *K. phaffii* NRRLY–11430 cells was performed using the electroporation method. For this purpose, 80 µL of the competent cells was mixed with the linearized DNA (~20 µg) into an ice-cold 0.2 cm electroporation cuvette (Bio-Rad Laboratories, Inc., Hercules, CA, USA). Electroporation was carried out by using the BioRad^®^ electroporation system at 1200 Volts, 200 Ω. After electroporation, 1 mL of 1 M sorbitol was added to the cuvette. The electroporated cells were transferred to a 15 mL tube and incubated at 30 °C for 2 h without agitation. After incubation, 100 and 200 µL were seeded onto YPDS plates (YPD medium supplemented with 1 M sorbitol) containing 100 µg/mL zeocin. The plates were incubated between 3 and 5 days at 30 °C, until colonies were observed. Colonies were later passed to 200 µg/mL zeocin YPDS plates.

### 4.4. Confirmation of Cassette Expression into K. phaffii

The insertion of the genes into *K. phaffii* NRRLY-11430 was confirmed using colony PCR. Reverse primers had complementary sequences with the BPs, while forward primers were specifically designed to identify the α-factor secretion signal (Table 3). The samples were prepared by mixing a portion of the colony with 3 µL of Zimolyase (5 μ/μL), incubating for 10 min at 30 °C, and then for an additional 10 min at −80 °C.

### 4.5. Expression of Recombinant Blocking Peptides

The evaluation of the clones was initially conducted at a 10 mL scale, in accordance with protocols that had been previously standardized at the IEIM [33,35]. Subsequently, the most productive clones for each BP were scaled up to 100 and 400 mL [35]. In brief, 1 mL of the glycerol vial from the working bank was cultured in YPD for 24 h at 30 °C and 250 rpm. Following the incubation period, the inoculum was added to BMGY medium (potassium phosphate buffer 100 mM, pH 6.0; yeast nitrogen base YNB 1.34% *w*/*v*; biotin 4 × 10^−5^% *w*/*v*, and glycerol). The culture was then incubated for 24 h at 30 °C and 250 rpm in shake flasks, until a DO_610nm_ between 2 and 6. The cells were harvested via centrifugation at 3500 rpm for 15 min, after which the pellet was resuspended in BMMY medium (potassium phosphate buffer 100 mM, pH 6.0; YNB 1.34% *w*/*v*; biotin 4 × 10^−5^% *w*/*v*, and methanol 0.5%). The culture was incubated for 72 h at 30 °C and 250 rpm, with methanol added at 12 h intervals to maintain a final concentration of 0.5% (*v*/*v*) during the 96 h induction period. The *K. phaffii* NRRLY-11430 wild-type strain was used as a negative control and incubated under identical culture conditions. For evaluation, 5% of the volume was sampled at 24 h intervals and stored at −20 °C for subsequent analysis.

The production of the recombinant BPs at a 1.65 L scale was carried out as previously described [31,33]. Cultures were prepared in a modified FM22 saline medium (composition per liter: 25.74 g KH_2_PO_4_, 3g (NH_4_)_2_SO_4_, 8.58 g K_2_SO_4_, 0.6 g CaSO_4_ 2H_2_O, 40 g glycerol, 7.02 g MgSO_4_ 7H_2_O, 4 × 10^−5^% biotin, 1 mL mineral traces, and 5 mL silicone). Protein production was performed in three phases: (i) a batch culture with glycerol to achieve 40 g/L biomass, (ii) a fed-batch culture with glycerol to achieve 60 g/L biomass, and (iii) a fed-batch induction phase with methanol concentration maintained at 0.5 ± 0.005% (*v*/*v*) with an automatic feed control. Cultures were carried out at pH 5.0 (maintained with 7% (*w*/*v*) ammonium hydroxide), 28 °C, and under limited oxygen conditions for 96 h.

### 4.6. Protein Purification

The supernatant was obtained after *K. phaffii* culture centrifugation at 4 °C and 10,000 rpm, followed by filtration through 0.45 and 0.22 µm membranes (Sartopore 2, Sartorius, Goettingen, Germany). For BP purification, a nickel affinity chromatography method was employed using the HisTrap^TM^ FF column (GE Healthcare, Piscataway, NJ, USA) on the ÄKTA pure™ system (GE Life Sciences, Piscataway, NJ, USA). Briefly, the culture supernatant was mixed with 2 mM MgSO_4,_ and pH was adjusted to 7.4. The Ni-NTA Sepharose column was equilibrated with binding buffer (20 mM sodium phosphate, 0.5 M NaCl, and 40 mM imidazole, pH 7.4). After loading the sample onto the column and performing the washing steps with binding buffer, the recombinant protein was eluted using the elution buffer (20 mM sodium phosphate, 0.5 M NaCl, and 500 mM imidazole, pH 7.4). Fractions with the highest absorbance at 280 nm and dot blot confirmation were pooled. Salts were removed using a 2 kDa Slide-A-Lyzer 2k dialysis cassette (Thermo Fisher Scientific Inc., Waltham, MA, USA) against PBS 1× buffer (8 g/L NaCl, 0.2 g/L KCl, 1.44 g/L Na_2_HPO_4_, and 0.24 g/L NaH_2_PO_4_).

### 4.7. Dot Blot for 6x-His Tag

To monitor the production and purification of BPs, a standardized dot blot immunodetection assay was performed. The dot blot analysis was conducted using a nitrocellulose blotting membrane (Protran NC, Cytiva, Amersham, UK). The adsorption of the crude extract or the purified BP adsorption was achieved by spotting 30 μL of each one. The membrane was left to dry for 1 h and blocked with 5% nonfat dry milk. After washing with TBS-T buffer (20 mM Tris-Base, 500 mM NaCl and 0.2% *v*/*v* Tween 20), the membrane was incubated with an anti-6X-His tag antibody 1:5000 (ab9108, Abcam, Cambridge, UK). The membranes was subsequently incubated with anti-rabbit immunoglobulin HRP conjugated 1:2000 (ab205718, Abcam, Cambridge, UK). The samples were exposed to a diaminobenzidine solution (0.1% imidazole, 0.01% diaminobenzidine, and 30% H_2_O_2_ in PBS 1X and 0.1% Triton X-100). The positive control for this assay was a confirmed histidine-labeled thermolabile esterase (E.C. 3.1.1.13) from *Acidicaldus* sp. produced in *E. coli* (a kind gift of Dr. Gina Mendez, USBA, Pontificia Universidad Javeriana) [76].

### 4.8. SDS-PAGE and Western Blot

Sodium dodecyl sulfate–polyacrylamide gel electrophoresis (SDS-PAGE) was performed using an adapted Tris-glycine protocol [77] under both reducing and non-reducing conditions. Protein samples (15 μL) were loaded onto 12% acrylamide resolving gels and 5% stacking gels in a Mini-PROTEAN electrophoresis system (Bio-Rad, Hercules, CA, USA). A ProSieve QuadColor protein marker (No. 00193837, Lonza, Basel, Switzerland) was used. The gel was stained with Coomassie Brilliant Blue R-250. For the Western blot immunoassay, the protein electrophoresis gel was transferred to a nitrocellulose blotting membrane (Protran NC, Cytiva, Amersham, UK) using a Bio-Rad Mini Trans-Blot Cell. The membrane was blocked with 5% nonfat dry milk, and proteins were detected via incubation with anti-6x-His tag antibody 1:5000 (ab9108, Abcam, Cambridge, UK). The membrane was then incubated with anti-rabbit immunoglobulin HRP conjugated 1:2000 (ab205718, Abcam, Cambridge, UK). Specific bands were visualized using a diaminobenzidine solution (0.1% imidazole, 0.01% diaminobenzidine, and 30% H_2_O_2_ in PBS 1× and 0.1% Triton X-100). The positive control used was a histidine-tagged recombinant thermolabile esterase (E.C. 3.1.1.13), as previously described [76].

### 4.9. Biomass Determination

Samples were used to evaluate the biomass present in the cultures during the induction time. Cell density was measured at 610 nm (DO_610_) using a BioSpectrometer Basic spectrophotometer (Eppendorf, Hamburg, Germany) using dilutions in water. BMMY culture medium was used as a blank. Biomass was calculated using a previously reported equation [30].

### 4.10. Protein Quantification

Protein quantification was performed using the Lowry method, with a Bovine Serum Albumin (BSA) standard ranging from 0.0625 to 2 mg/mL. Protein quantification was also performed via densitometric analysis with ImageJ 1.53a [78]. Protein samples were evaluated using SDS-PAGE, as described above. After electrophoresis, the gel was stained with Coomassie Brilliant Blue to visualize the protein bands. For quantification, a standard curve was generated with BSA concentrations ranging from 0.0625 to 0.25 mg/mL. The gel was scanned to create a digital image, which was subsequently analyzed using ImageJ 1.53a [78].

### 4.11. Determination of BPs Binding with RBD by Dot Blot

To determine the binding of recombinant BPs with RBD variants, the experiments were performed at the Unidad de Desarrollo e Investigación en Bioterapéuticos (UDIBI), Escuela Nacional de Ciencias Biológicas (Ciudad de México, Mexico). Purified fractions of BPs were fixed onto a nitrocellulose blotting membrane (Protran NC, Cytiva, Amersham, UK) after blocking with PBS 1×, 5% nonfat powdered milk, and the RBD (1 µg/mL) of SARS-CoV-2 variants was added. Variants evaluated included beta, delta, delta plus, BA1, BA 4/5, XBB, and BQ 1.1 provided by UDIBI. An anti-RBD antibody produced in UDIBI (2 µg/mL), in a blocking solution, was used as the primary antibody. The membrane was washed with PBS 1×/Tween 20 0.1%, and a human anti-IgG antibody conjugated with an HRP enzyme 1:15,000 (ab97225, Abcam, Cambridge, UK) was added. The assay was developed using ECL™ Prime Western Blotting Detection Reagent (Thermo Fisher Scientific Inc., Waltham, MA, USA) and the signal emission was read using a ChemiDoc MP Imaging System (Bio-Rad Laboratories, Inc., Hercules, CA, USA). BSA was used as the negative control. Dot blot results were analyzed using densitometry with ImageJ 1.53a [78].

### 4.12. Blocking Assay

The blocking capacity of BPs was evaluated using the CPass SARS-CoV-2 Neutralization Antibody Detection Kit (GenScript, Piscataway, NJ, USA). The assay evaluates the binding competency of the RBD-HRP protein to hACE2 in the presence of potential inhibitory molecules present in the samples. The assays allowed us to evaluate the blocking by interaction with SARS-CoV-2 RBD variants of Wuhan, Mu BA.1, and MU BA.2. Briefly, the RBD was allowed to interact with the BPs for 30 min at 37 °C. BP2 concentrations of 121.17, 60.59, 30.29, 15.15, 7.57, 3.79, and 1.89 ng/µL were evaluated; while BP9 concentrations of 226.80, 113.40, 56.70, 28.35, 14.20, 7.08, and 3.54 ng/µL were evaluated. Then, 100 µL of the reaction was added to the hACE2-coated assay plate and led for reaction at 37 °C for 15 min. The plate was washed with a 1X wash solution. 3,3′,5,5′ tetramethylbenzidine (TMB) was used as an HRP substrate and led to react in the dark for 25 min at room temperature. Results were expressed as a percentage of inhibition. IC_50_ was estimated using a nonlinear regression with GraphPad Prism 8.0. The “Absolute IC50, X is concentration” equation was selected, and the top and baseline values were set to 100 and 0, respectively.

## 5. Conclusions

This study demonstrates the potential of blocking peptides produced in *K. phaffii*, designed to inhibit the interaction between RBD and hACE2, to bind to SARS-CoV-2 RBD from Beta, BA.4/5, Delta, Delta+, XBB, BQ1.1, and BA.1 variants. These peptides have the capacity to block the interaction between hACE2 and the RBD of the Wuhan, MU, BA.1, and BA.2 variants. The protein production process was scaled up to 400 mL and 1.65 L, resulting in a higher yield of production at the 96 h culture stage. Despite losses during affinity chromatography and desalting, the BPs were ultimately obtained with yields of 14.3 mg/L for BP2, 4.01 mg/L for BP9, and 1.35 mg/mL for BP11. The production of BP2 was found to be greater than that of BP9 and BP11. Notably, both BP2 and BP9 demonstrated IC_50_ values in the nM range, which is comparable to those reported for other peptides designed to block the RBD and hACE2 interaction [15,49,52]. Based on its production yield, binding affinity, and blocking efficacy, BP2 stands out as a promising candidate for a novel therapeutic approach to treat SARS-CoV-2 infection. This is especially pertinent for the immunodeficient population, who are disproportionately affected by SARS-CoV-2 and for whom current emergency treatments, such as monoclonal antibodies, are losing efficacy [79,80,81,82]. Therefore, further research is needed to evaluate the safety and effectiveness of the BPs characterized in this study.

## Figures and Tables

**Table 1:** Predicted physicochemical properties BP2, BP9, and BP11.

Peptide	Number of Amino Acids	Molecular Weight (Da)	Theoretical *Ip*	Ext. Coefficient	Grand Average of Hydropathicity (GRAVY)
BP2	118	14,832.43	5.68	22,585	−0.666
BP9	132	16,532.33	5.55	24,075	−0.656
BP11	132	16,532.33	5.55	24,075	−0.656

**Table 2:** BPs quantification and production yield at 1.65 L scale. Proteins were quantified using densitometric method.

Blocking Peptide	Protein (mg)	Volumetric Productivity (mg per Culture L)	Protein Production mg of Protein per Biomass (mg/g)	Productivity Yield (mg/L·h)
BP2	23.66	14.34	8.72	0.15
BP9	6.62	4.01	8.03	0.04
BP11	2.23	1.35	1.44	0.01

**Table 3:** List of primers used to confirm the *K. phaffii* NRRLY-11430 clones.

Primer	Sequence	Amplicon Length (bp)
Forward BP	5′-CGAGAAAAGAGAGGCTGAAGC-3′	--
Reverse BP2	5′-CAATGGGTACATCTGAGCCAAAG-3′	427
Reverse BP9	5′-AGAAGATTGGTAGAACAAGTCCTCG-3′	307
Reverse BP11	5-AGAAGATTGGTAGAACAACTCGTCG-3′	307

## Data Availability

Dataset available upon request from the authors.

## Supplementary Materials

The following supporting information can be downloaded at https://www.mdpi.com/article/10.3390/ph17091240/s1. Table S1: Additional physicochemical properties predicted for BPs. Table S2: Data from bioinformatic analysis on BPs-RBD interactions. Figure S1: Expression cassette inserted in BP9 (A) and BP11 (B) clones were confirmed via DNA sequence. Figure S2: Representative chromatograms of the purification process from the 1.65 L cultures. Figure S3: N-glycosylation prediction for BP2, BP9, and BP11.
