# Supplementary material for: Study of Potential Blocking Peptides Targeting the SARS-CoV-2 RBD/hACE2 Interaction
Source: Pharmaceuticals (Basel). 2024 Sep 20;17(9):1240. doi: 10.3390/ph17091240 (PMC11435355; doi:10.3390/ph17091240)
Supplement: Supplementary file 1 [file pharmaceuticals-17-01240-s001.zip › pharmaceuticals-3214483-supplementary.pdf]

Supplementary Materials

Study of Potential Blocking Peptides Targeting the SARS-CoV-2 RBD/hACE2 Interaction

Sara M. Villada-Troncoso, Jenny Andrea Arévalo-Romero, Vanessa Hernández Rivera, Martha Pedraza-Escalona, Sonia M. Pérez-Tapia, Angela Johana Espejo-Mojica and Carlos Javier Alméciga-Díaz

Table of content

Supplementary Table S1. Additional physicochemical properties predicted for BPs.......................... 3

Supplementary Table S2. Data from bioinformatic analysis on BPs-RBD interactions........................ 4

Supplementary Figure S1. Expression cassette inserted in BP9 (A) and BP11 (B) clones were confirmed by DNA sequence. 5

Supplementary Figure S2. Representative chromatograms of the purification process from the 1.65 L cultures... 6

Supplementary Figure S3. N-glycosylation prediction for BP2, BP9 and BP11..................................... 7

Supplementary Table S1. Additional physicochemical properties predicted for BPs.

Property	BP2	BP9	BP11
Estimated half-life	20 hours (mammalian reticulocytes, *in vitro*). 30 min (yeast, *in vivo*). >10 hours (*Escherichia coli, in vivo*)	7.2 hours (mammalian reticulocytes, *in vitro*). > 20 hours (yeast, *in vivo*). >10 hours (*Escherichia coli, in vivo*)	7.2 hours (mammalian reticulocytes, *in vitro*). > 20 hours (yeast, *in vivo*). >10 hours (*Escherichia coli, in vivo*)
Instability index	52.47	60.06	57.96
Aliphatic index	67.03	70.30	70.30

Supplementary Table S2. Data from bioinformatic analysis on BPs-RBD interactions.

**BP**	**ΔG (kcal/mol)**	**K_d (M)**	**ICs charged-charged**	**ICs charged-polar**	**ICs charged-apolar**	**ICs polar-polar**	**ICs polar-apolar**	**ICs apolar-apolar**	**NIS charged**	**NIS apolar**
BP2	-12.1	1.3×10^9	3	7	13	6	23	13	19.11	36.52
BP9	-10.6	1.6×10^8	3	5	7	6	18	18	19.87	34.68
BP11	-11.4	4.6×10^9	8	13	25	3	10	21	18.84	36.99

ICs: Number of contacts at the interface

NIS: Non-interacting surfaces

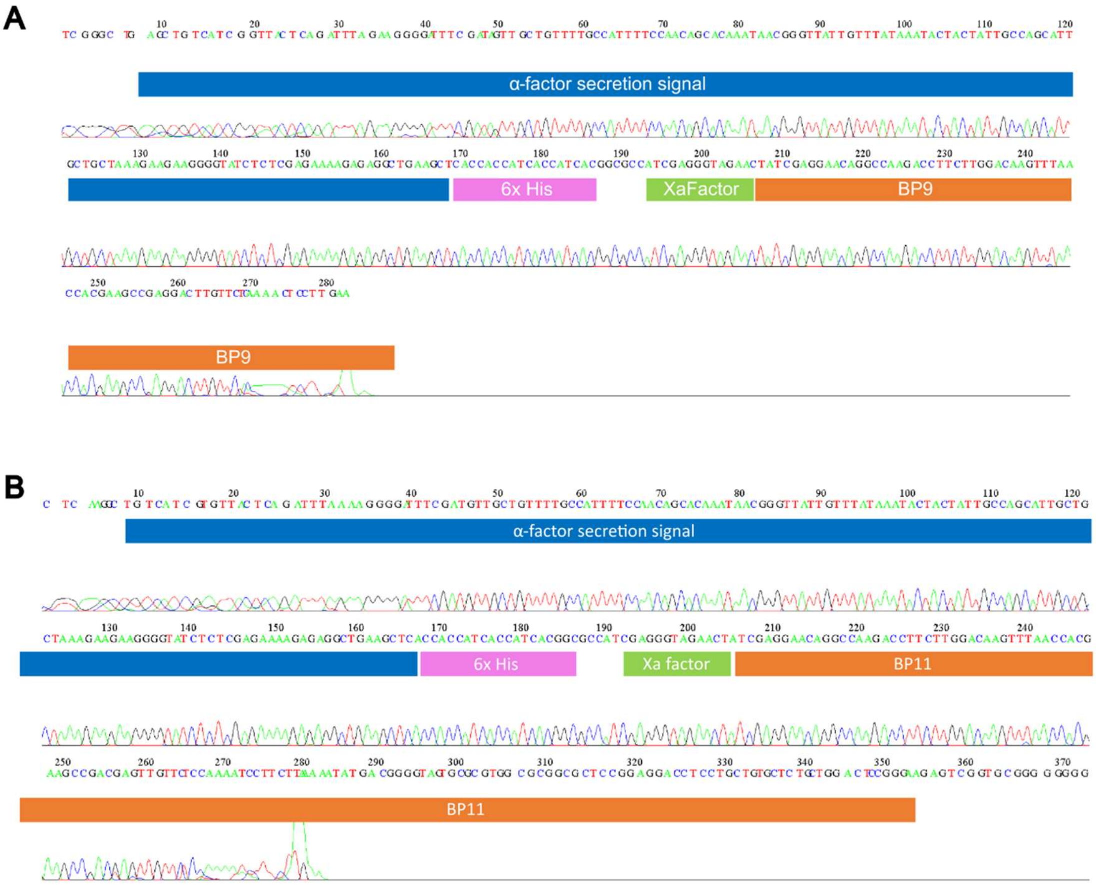

Supplementary Figure S1. Expression cassette inserted in BP9 (**A**) and BP11 (**B**) clones were confirmed by DNA sequence. Sequencing results showed the presence of the α-factor secretion signal, 6x-His tag , Xa factor, and a fragment of the BPs sequence.

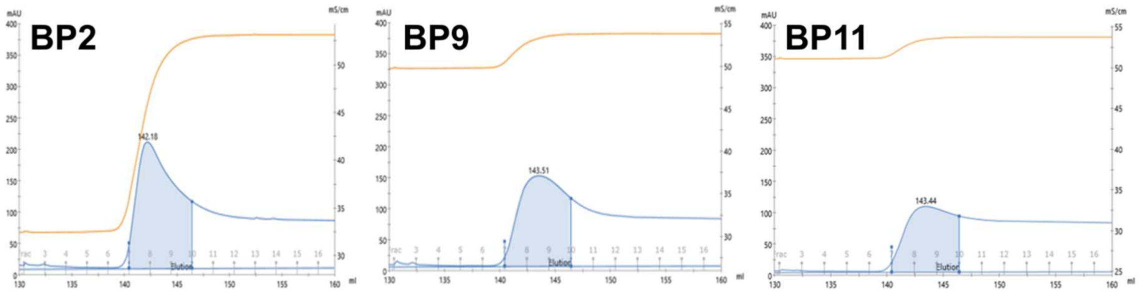

Supplementary Figure S2. Representative chromatograms of the purification process from the 1.65 L cultures. The supernatant was obtained by centrifugation at 4 °C and 10000 rpm, followed by filtration through 0.45 and 0.22 μm membranes. For BP purification, a nickel affinity chromatography method was employed using the HisTrapTM FF column (GE Healthcare, Piscataway, NJ) on the ÄKTA pure™ system (GE Life Sciences, Piscataway, NJ).

A

```
Name:  Sequence         Length:  118
IEEQAKTFLDKFNHEAEDLFYQSSLASWNYNTNITEENVQNMNNAGDKWSAFLKEQSTLAQMYPLQEIQNLTVKLQNGTI      80
YSTGTQGFSENSMLTSKAVCHPTAWDLGKGDFRILMCT                                               160
................................N....................................N......N...      80
......................................                                               160

(Threshold=0.5)
----------------------------------------------------------------------
SeqName      Position  Potential   Jury    N-Glyc
                                 agreement result
----------------------------------------------------------------------
Sequence       33 NITE   0.7527     (9/9)    +++
Sequence       70 NLTV   0.6559     (9/9)    ++
Sequence       77 NGTI   0.6338     (7/9)    +
----------------------------------------------------------------------
```

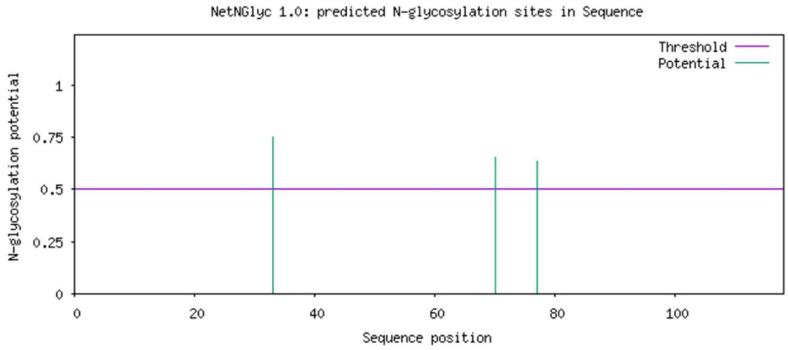

B

```
Name:  Sequence         Length:  132
TIEEQAKTFLDKFNHEAEDLFYQSSLASWNYNTQITEENVQNMNNAGDKWSAFLKEQSTLAQMYPLQEIQQLTVKLQLQA      80
LQQNSGSGAVCHPTAWDLGKGDFRILSCTKVTMDDFLTAHHEMGHIQYDMAY                                 160
................................................................................      80
....................................................                                 160

(Threshold=0.5)

No sites predicted in this sequence.
```

C

```
Name:  Sequence         Length:  132
TIEEQAKTFLDKFNHEADELFYQSSLASWNYNTQITEENVQNMNNAGDKWSAFLKEQSTLAQMYPLQEIQQLTVKLQLQA      80
LQQNSGSGAVCHPTAWDLGKGDFRILSCTKVTMDDFLTAHHEMGHIQYDMAY                                 160
................................................................................      80
....................................................                                 160

(Threshold=0.5)

No sites predicted in this sequence.
```

Supplementary Figure S3. N-glycosylation prediction for BP2, BP9 and BP11. Prediction of N-glycosylation sites within BP2 (**A**), BP9 (**B**), and BP11 (**C**) sequences was carried by using the NetNGlyc 1.0 server. Asn-Xaa-Ser/Thr sequons in the sequence output below are highlighted in blue. Asparagines predicted to be N-glycosylated are highlighted in red. A position with a potential (vertical lines) crossing the threshold (horizontal line at 0.5) is predicted glycosylated.
